# Dual, High and Worsening Burden of Malnutrition Among Under‐5 Children Living in Malawi's Cities: Evidence From the 2015/16 and 2024 Demographic and Health Surveys

**DOI:** 10.1002/fsn3.71581

**Published:** 2026-02-27

**Authors:** Alexander A. Kalimbira, Patrick Singoyi, Gareth Osman, Doris C. Nanga, Khumbo Mhango, Bridget Mkama, Numeri C. Geresomo, Zione Kalumikiza‐Chikumbu

**Affiliations:** ^1^ Department of Human Nutrition and Health Lilongwe University of Agriculture and Natural Resources Lilongwe Malawi

**Keywords:** cities, Demographic and Health Survey, Malawi, overweight, stunting

## Abstract

This study examined the structure of, relationship between, and changes in the burden of undernutrition (stunting) and overnutrition (overweight) among children under the age of 5 years living in the cities of Blantyre, Lilongwe, Mzuzu, and Zomba in Malawi. We analyzed the prevalence of undernutrition and overnutrition from the 2015/16 Malawi Demographic and Health Survey (MDHS), which we compared with anthropometric results of the 2024 MDHS that were published in the Key Indicators Report released in February 2025. We calculated the prevalence of moderate stunting and the contribution of severe stunting and moderate stunting to overall stunting in each city. Anthropometric results were available for a weighted sample of 256 children in 2015/16 and 351 children in 2024. Compared to 2015/16, prevalence of stunting and overweight increased in all cities, with Lilongwe, the capital, recording the largest increase in both stunting (86.7%) and overweight (118.6%). Except for Mzuzu city, which experienced a *high* prevalence of stunting (24.7%), the prevalence of stunting and overweight were *very high* (range 31.1%–41.5%) in the other three cities in 2024. Regarding overweight, prevalence ranged from *medium* (5 to < 10%) in Blantyre and Lilongwe to *high* (10 to < 15%) in Mzuzu and Zomba. The contribution of severe stunting to overall stunting more than quadrupled from 7% in 2015/16 to 30.6% in 2024 in Lilongwe. We conclude that children who live in Malawi's four cities have a dual burden of malnutrition, with *very high* prevalence of stunting and *medium* to *high* prevalence of overweight. Most of the children are moderately stunted. Nutrition policies and strategies for city dwellers should be formulated and implemented to improve nutrition. A larger study that aims to understand the structure and determinants of malnutrition in the cities is required.

## Graphical abstract

Stunting and overweight prevalence among under five children rose across all four Malawian cities between 2015/16 and 2024, with the capital city registering the most dramatic increase of 86.7% and 118.6% respectively. By 2024, stunting prevalence moved from *low* to *high* for Lilongwe and Zomba, *low* to *medium* for Mzuzu, and, *high* to *very high* for Blantyre. Overweight increased by about 2‐fold in Blantyre and Lilongwe, with, 2.7 and 6.1 percentage point increase in Mzuzu and Zomba, respectively. Overall, stunting increased from 20.8% to 37.6%, and overweight from 6.1% to 10.3% over the period.

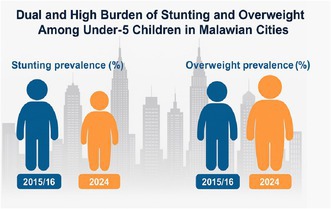

## 1 Introduction

Malawi, a country often associated with rural poverty (National Statistical Office 2022), food insecurity (Integrated Food Security Phase Clasification (IPC) 2024) and undernutrition (Government of Malawi 2018) is now facing a new crisis in its cities, the dual burden of malnutrition (National Statistical Office ICF 2024; National Statistical Office 2022), manifesting in the form of stunting on the one hand, and overweight on the other.

In Sub‐Saharan Africa, urban malnutrition is characterized by a coexistence of stunting, wasting, and overweight among children. A study in Ghana found that urban children were more likely to be overweight compared to their rural counterparts, with a prevalence of 8.3% in urban versus 3.2% in rural areas (Frempong and Annim 2017). In Kenya, 40% of children living in Nairobi's slums were stunted, driven by poor sanitation, food insecurity, and limited access to healthcare (Kimani‐Murage et al. 2015). In Southeast Asian countries like Indonesia and the Philippines, urban children were more likely to be overweight due to consumption of energy‐dense, nutrient‐poor foods (Popkin et al. 2012). However, stunting remains prevalent, particularly among the urban poor, with 36% of urban children in Indonesia being stunted and higher rates observed in low‐income households (Rachmi et al. 2017). In South America, Monteiro and colleagues reported a significant increase in overweight and obesity among urban children in Brazil, with prevalence rates reaching 15% in some cities (Monteiro et al. 2004). Conversely, undernutrition persists in urban slums, with 20% of children in urban areas being stunted, particularly in informal settlements where poverty and food insecurity are prevalent (Victora et al. 2008).

Urbanization reshapes food environments, posing challenges to access affordable, healthy food options by the urban populations, especially vulnerable groups such as children and low‐ income workers (Ameye et al. 2025). The transition of countries to middle‐income status contributes to nutrition transition due to increased availability, accessibility of ultra‐processed foods and beverages which are also convenient and affordable, hence potentially increasing the prevalence of overweight among children across all household income levels (UNICEF 2025). The rapid growth in middle income countries has been regarded as a key factor to the rising global sales of ultra‐processed foods and beverages where modern retail outlets, online grocery stores and food delivery apps are expanding rapidly, alongside traditional outlets. (UNICEF 2025). Ameye et al. (2025) underscores that consumption of ultra‐processed foods is larger in urban than rural areas of Africa. Current data on the new indicators for monitoring the consumption of unhealthy foods and beverages in children aged 6–23 months show a 50% consumption of sweet, salty and fried foods and sweet beverages (UNICEF 2025). A study in one of the urban settings in Malawi revealed a daily consumption of ultra‐processed foods particularly those high in sugar among school going children (Kamanga et al. 2024). This could be considered as a result of the unhealthy food environments which are affecting nutritious foods choices over convenience posing threats to increased childhood malnutrition.

Malawi's urbanization aspiration aims to increase the urban population from 17.1% of the total population in 2019 to 60% by 2063 (National Planning Commission 2020). In recognition of socioeconomic challenges in slums, the country plans to reduce the proportion of the urban population living in slums or inadequate housing from 60% in 2020 to 10% by 2063. Additionally, the vision calls for the creation of secondary cities to decongest major urban centers.

Rapid urbanization (e.g., the 5% increase that Malawi is facing, which outpaces the development of essential infrastructure and services) can become an important recipe for high rates of malnutrition if social issues, including rising poverty, unemployment, and inequality are not timely addressed (National Planning Commission 2020). Based on experiences from other developing countries, urbanization comes with significant public health challenges. Despite being linked to economic growth and improved living standards, the assumption that urban areas are nutritionally secure is increasingly being challenged as new evidence suggests that urban malnutrition is not only significant, but in some cases worse off than in rural areas (Vilar‐Compte et al. 2021). Our study draws results from two successive Malawi Demographic and Health Surveys (MDHSs): the 2015/16 MDHS and the 2024 MDHS, both of which disaggregated malnutrition data at the city level. We aimed to examine the structure and interrelationship of indicators of undernutrition, stunting and overweight, among children under the age of 5 years living in the cities of Blantyre, Lilongwe, Mzuzu, and Zomba in Malawi over two survey periods.

## 2 Methods

### 2.1 Description of the 2015/16 and 2024 MDHSs

We used cross‐sectionally generated secondary data from the 2015/16 and 2024 MDHSs implemented by the National Statistical Office (NSO) of Malawi (NSO and ICF 2024, 2017). The DHSs are large‐scale nationally representative surveys that provide comprehensive data on population, health, and nutrition indicators. Data collection is conducted through face‐to‐face interviews, with questionnaires administered to women of reproductive age (15–49 years), men (15–54 or 15–59 years), and households. The surveys cover a wide range of topics, including fertility, family planning, maternal and child health, nutrition, and socioeconomic characteristics, and therefore become a valuable resource for public health research and policy planning. Specifically, the 2024 MDHS was also conducted to provide data relevant to the Sustainable Development Goals for Malawi.

### 2.2 Sample Size, Sampling Frame and Sampling Procedure

In 2015/16, a total of 26,361 households were successfully interviewed and anthropometric data (height and weight) were available for 5233 eligible children under the age of 5 years. In the 2024 survey, 22,414 households were successfully interviewed, and 4187 children under the age of 5 years were eligible for anthropometric measurements. The sampling frame used for the 2015/16 survey was the 2008 Malawi Population and Housing Census (National Statistical Office (NSO) [Malawi] ICF 2017) in which a stratified two‐stage cluster sampling design was used to ensure national and subnational representativeness. In the first stage, clusters were selected using probability proportional to size for urban and rural areas in each of the 28 districts and the 4 cities of Mzuzu in the Northern region, Lilongwe in the Central, and Blantyre and Zomba in the Southern region of Malawi. In the second stage, households were randomly selected from updated lists within the chosen clusters. A similar procedure was followed in the 2024 MDHS, but using an updated sampling frame based on Malawi's 2018 Population and Housing Census (National Statistical Office ICF 2024). The representative samples for 2015/16 and 2024 survey were 256 and 351, respectively for all four Malawian cities.

### 2.3 Assessment of Nutritional Status

The NSO collected anthropometric data from children under the age of 5 years and reported that the resulting Z‐score computations used to assess nutritional status were based on the 2006 World Health Organization (WHO) child growth standards (WHO Multicenter Growth Reference Study Group 2006). Stunting was defined as height‐for‐age Z‐scores below −2 standard deviations of the median in the WHO child growth standards, while overweight was defined as a weight‐for‐height Z‐score above +2 standard deviations of the median.

### 2.4 Data Analysis

The sample for the four cities was drawn from the MDHS for both the 2015/2016 and 2024 survey. For 2015/2016 MDHS, city level data were available in the dataset but not reported in the published reports. Stata version 17 was therefore used to re‐analyze the 2015/2016 MDHS data, from which data for the four cities and length‐for‐age Z‐score variables were extracted. Children with height‐for‐age Z scores below −2 were categorized as stunted while those with Z scores below −3 were categorized as severely stunted. Children with Z‐score above +6 and below −6 were excluded in analysis in accordance with WHO data quality guidelines (WHO Anthro for Personal computers 2011). The prevalence of stunting and severe stunting was then estimated using descriptive frequency percentages, with adjustments for survey sampling weights to ensure comparability with the 2024 MDHS estimates. Because raw data for 2024 were not yet available by the time this paper was written, we used results published in Table 12 of the Key Indicators Report (National Statistical Office ICF 2024), which we compared to 2015/16 to visualize the structure of malnutrition in the four cities.

The MDHS reports do not report on moderate malnutrition, which was important in our study. Therefore, Microsoft Excel was used to calculate both the prevalence of moderate stunting and the proportional contribution of moderate and severe stunting to the overall prevalence of stunting. The prevalence of moderate stunting was calculated by subtracting the prevalence of severe stunting from the overall prevalence of stunting. For the proportional analysis, the proportional contribution of severe stunting to the overall prevalence of stunting was calculated by dividing the prevalence of severe stunting by the overall prevalence of stunting, then multiplying the result by 100 to express it as a percentage. Since severe stunting (height‐for‐age Z‐score < −3 SD) is a subset of overall stunting (height‐for‐age Z‐score < −2 SD), the percent contribution of moderate stunting was derived by subtracting the contribution of severe stunting from 100%. Thus:

$$\text{Severe Stunting Contribution}\;\left(\%\right)=\left(\frac{\text{Prevalence of Severe Stunting}\;\left(<-3\mathrm{SD}\right)}{\text{Overall Prevalence of Stunting}\;\left(<-2\mathrm{SD}\right)}\right)\times 100$$

Moderate stunting contribution%=100%−Severe stunting contribution%

Graphs were used to compare the prevalence of moderate and severe stunting, as well as the proportional contributions of severe and moderate stunting to visualize the distribution and structure of malnutrition across the four cities. Descriptive statistics were appropriate because both surveys were nationally representative and were conducted using the same DHS sampling design, data collection procedures, and anthropometric measurement standards. However, no formal statistical test of differences was performed, as this was not possible: the 2024 MDHS survey data were only available in published summary tables, while the 2015/2016 MDHS data were reanalyzed from the raw dataset.

## 3 Results

### 3.1 Sample Sizes and Mean Z‐Scores for Height‐For‐Age, Weight‐For‐Height, and Weight‐For‐Age Among Children Living in Malawian Cities

Although anthropometric results were available for 3796 from the 2024 MDHS, the city sample was 351, representing 9.2%. Table 1 compares prevalence of different forms of malnutrition as estimated in the 2015/16 and 2024 MDHSs.

**TABLE 1:** Prevalence of stunting and overweight among children under the age of 5 years living in Malawi's cities in 2015/16 and 2024.

City	Stunting					Overweight	
	% Stunted			% Severely Stunted		% Overweight	
	2015/16	2024		2015/16	2024	2015/16	2024
Blantyre	30.5	41.5		5.2	9.1	3.2	6.2
Lilongwe	19.6	36.6		1.4	11.2	4.3	9.4
Mzuzu	15.4	24.7		4.5	9.3	8.5	11.2
Zomba	17.8	31.1		5.3	5.6	8.4	14.5
All	20.8	37.6		3.5	11.6	6.1	10.3

In 2024, prevalence of stunting was highest (41.5%) in Blantyre city and lowest (24.7%) in Mzuzu city. Except for Mzuzu city, the prevalence of stunting in the other three cities was *very high* based on thresholds for classifying childhood malnutrition (de Onis et al. 2019). Regarding overweight, the prevalence ranged from medium (5 to < 10%) in Blantyre and Lilongwe to *high* (10 to < 15%) in Mzuzu and Zomba cities. There was very low (< 2.5%) prevalence of wasting, with no severely wasted children identified in all four cities (results not shown).

### 3.2 Rate of Increase in Prevalence of Stunting and Overweight

**FIGURE 1:**
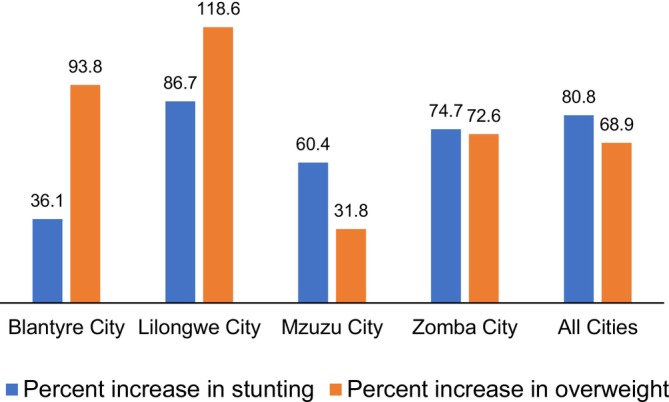
Percent increase between 2015/16 and 2024 in the prevalence of stunting and overweight among children under the age of 5 years living in Malawi's four cities.

A comparison between 2015/16 and 2024 revealed inter‐city variations in changes in prevalence of stunting and overweight (Figure 1). Children from the capital Lilongwe exhibited the highest relative increase in stunting (86.7%) and overweight (118.6%), which is the highest dual burden among the four cities. Blantyre depicted the smallest percent increase in stunting while Mzuzu depicted the smallest increase in overweight. Blantyre had the widest difference between increased prevalence of stunting (36.1%) and increased prevalence of overweight (93.8%). The percent increase in the prevalence of stunting and overweight was similar in Zomba city.

### 3.3 Burden of Severe and Moderate Stunting

**FIGURE 2:**
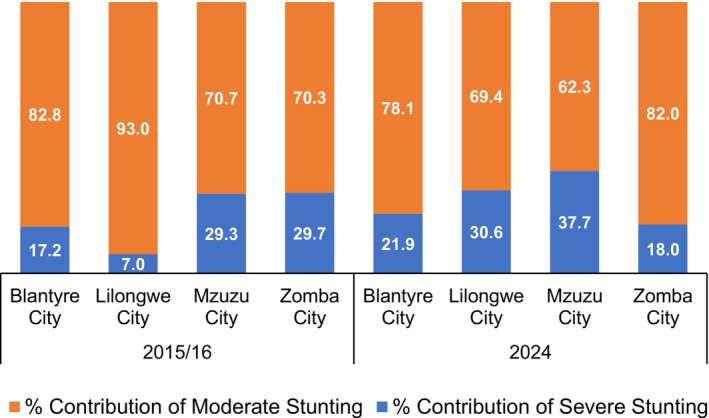
Proportionate contribution of severe and moderate stunting to overall burden of stunting among children under the age of 5 years living in Malawi's four cities.

Figure 2 shows the proportionate contribution of moderate and severe stunting to the overall prevalence of stunting. Moderate stunting was the dominant form of stunting in all cities, but more prominently in Lilongwe city in 2015/16 where more than 9 in 10 stunted children were moderately stunted. Except in Zomba city where the contribution of severe stunting declined by nearly 12 percentage points between 2015/16 and 2024, the proportion increased in the three other cities, with Lilongwe depicting the largest change of nearly 24 percentage points.

## 4 Discussion

The 2024 MDHS has revealed the existence of a dual burden of malnutrition in Malawi's four cities of Blantyre, Lilongwe, Mzuzu and Zomba. Until the 2024 MDHS, there was no certainty on the burden of malnutrition among Malawian children living in the cities of Blantyre, Lilongwe, Mzuzu and Zomba. Malnutrition has been well‐established in Malawi's “urban” areas since the MDHS of 1992, which disaggregated the prevalence of malnutrition by residence; however, there has been a lack of insights into the trends and magnitude of malnutrition over time, as demonstrated by this study. In Malawi, “urban” classification encompasses the four cities of Lilongwe, Blantyre, Mzuzu, and Zomba along with secondary urban centers, including townships and district capitals, whereas all remaining regions are designated as ‘rural’ (McBride and Moucheraud 2022). Albeit being small, the generation of a city sample has unveiled that children living in Malawi's cities are just as malnourished as their rural counterparts. This is not surprising, given that urban areas have many challenges such as food insecurity (Mkusa 2020), poor water and sanitation (Manda 2009), and inadequate housing (UN‐Habitat, n.d.) all of which are some of the well‐known determinants of stunting (Rahut et al. 2024); (Kassie and Workie 2020); (Wali et al. 2020).

A typical dual burden of malnutrition is evident in Malawian cities among children since there was a *very high* prevalence of stunting in three of the four cities, and a *high* prevalence in one, which coexisted with *medium* to *high* prevalence of overweight within the same population. These findings are similar to what is known about many regions of low‐ and medium‐income countries, where undernutrition and overnutrition coexist in the same population (Winichagoon and Margetts 2017). While urbanization is often expected to improve food access, factors such as high living costs, poor maternal nutrition, and urban food deserts could be driving malnutrition. Vilar‐Compte and others systematically reviewed the relationship between urban poverty and malnutrition, demonstrating that contrary to expectations, urban poverty is a significant driver of child malnutrition. They identified economic barriers, limited access to healthy foods, and food insecurity as key contributors to poor nutritional outcomes, including obesity and micronutrient deficiencies, among urban poor children. The authors challenged the assumed “urban advantage” in food access, and highlighted the need for targeted interventions to address urban nutritional inequities (Vilar‐Compte et al. 2021).

Comparing 2015/16 and 2024 results at national level, the prevalence of stunting and overweight among children below the age of 5 years has slightly gone up from the 2015/16 MDHS by two percentage points for stunting, and one percentage point for overweight (National Statistical Office ICF 2024). Even in the absence of preceding data, we do not expect that the prevalence rates observed in the four cities are an improvement, for there is nothing unique about the cities which would be a basis for that expectation. What is clear is that the *medium* to *high* prevalence of overweight and the *high* to *very high* prevalence of stunting highlight the need for policies and programs that address both under‐ and overnutrition in Malawi's cities. Lack of nutrition‐focused urban policies and programs would only exacerbate the vulnerability of children who live in these cities.

In both 2015/16 and 2024, moderate stunting exerts a large burden on the overall prevalence of stunting. What is concerning is that except for Zomba city, the proportion of severely stunted children rose between 2015/16 and 2024, suggesting a worsening situation. However, more data points would reveal if the increase was a one‐time occurrence or is systematic. At this point, it would be reasonable to suspect that the increase in the proportion of severely stunted children represents a reality since the overall prevalence of stunting has increased, and that the increase is evident in three of the four cities. Given that stunting reflects multiple socio‐economic failures at household, community, and national levels (World Health Organization 2016), severe stunting is therefore a manifestation of worsening failures. This means that conditions for child growth worsened between 2015/16 and 2024 in Malawi's cities.

With increasing urbanization, the situation can only be expected to worsen unless more evidence is generated and structured interventions are implemented to avert a more serious situation. Data is needed to understand the prevalence of malnutrition in different types of urban settlements: social, economic and demographic characteristics of households and communities in which malnutrition exists at a concerning magnitude, and the determinants of different forms of malnutrition. Furthermore, studies are needed to understand how food systems are shaping malnutrition among children living in cities; for example, the availability of food swamps and food deserts, and the contribution of other factors such as infant and young child feeding (IYCF) practices. Already, it is not known how caregivers who live in Malawi's cities access messages on IYCF since nutrition extension education is more visible in rural areas where frontline extension workers from the key sectors of agriculture and health are placed. Whether the care group model (Perry et al. 2015); (USAID Advancing Nutrition 2024), which the Government of Malawi promotes as a model for nutrition extension education (Government of Malawi 2021), would work in Malawi's city settings is a hypothesis that needs to be tested before scaling up and out.

The findings of this study have important implications for urban child health and nutrition policy in Malawi. The co‐existence of very high stunting and medium to high overweight prevalence among under‐five children in Malawi's four main cities underscores the urgent need for integrated, city‐specific nutrition interventions. Targeted interventions should address both undernutrition and the rising trend of overweight and obesity, particularly in rapidly urbanizing settings where structural challenges such as food insecurity, limited access to nutritious foods and health services, and poor sanitation often undermine child health and nutrition. Urban nutrition policies must prioritize vulnerable populations living in informal settlements and slums, where children are disproportionately affected by these conditions. This prioritization should include ensuring availability and access to affordable, nutritious foods and promoting optimal IYCF practices through context‐specific and tailored community‐based approaches. In addition, expanding nutrition education programs and extension services traditionally concentrated and intensified in rural areas into urban communities is essential for realizing Malawi's ambitious urbanization agenda and ensuring that urban growth leads to improvements in child health and nutrition outcomes.

The strength of our study lies in the use of nationally representative datasets based on a similar methodology that allows comparison over time. Another strength is that this is the first time the dual burden of malnutrition among children living in Malawian cities has been elucidated and quantified. However, we acknowledge limitations of the study. First, the lack of access to raw data for the 2024 MDHS limited our ability to establish associations between the burden of malnutrition and their underlying determinants in the cities. Second, the relatively small city‐level sample sizes, particularly in the 2015/16 MDHS, may affect the generalizability of the findings. Third, differences in data handling between the two datasets, where the 2015/2016 data were reanalyzed while the 2024 data were taken from published tables, may have introduced inherent errors in the 2024 MDHS. Future studies with larger sample sizes and longitudinal designs are needed to better understand the drivers and dynamics of malnutrition in Malawi's cities.

In conclusion, our findings reveal a rising dual burden of malnutrition among children under 5 years resident in the four cities in Malawi, which can only be expected to rise unless city‐context interventions are implemented. With the country aiming to increase its urban population as part of the long‐term ambition to create an inclusively wealthy and self‐reliant nation, Malawi's long‐term human capital development would be undermined without an expedited and deeper understanding of the coexistence of undernutrition (e.g., stunting), and overnutrition (e.g., overweight). We recommend that stakeholder dialogue be organized as a platform for designing urban nutrition strategies and by‐laws on key areas such as urban markets, school environments, restaurants and fast foods shops.

## Author Contributions

**Alexander A. Kalimbira:** conceptualization (lead), data curation (supporting), formal analysis (lead), methodology (equal), resources (equal), software (equal), supervision (equal), validation (equal), writing – original draft (equal), writing – review and editing (equal). **Patrick Singoyi:** data curation (lead), formal analysis (lead). **Gareth Osman:** validation (equal), writing – original draft (supporting), writing – review and editing (supporting). **Doris C. Nanga:** conceptualization (supporting), project administration (supporting), validation (equal), visualization (equal), writing – original draft (supporting). **Khumbo Mhango:** validation (equal), writing – original draft (equal), writing – review and editing (equal). **Bridget Mkama:** data curation (equal), formal analysis (equal), validation (equal). **Numeri C. Geresomo:** conceptualization (equal), supervision (lead), validation (equal), writing – review and editing (supporting). **Zione Kalumikiza‐Chikumbu:** conceptualization (equal), data curation (equal), formal analysis (supporting), methodology (equal), project administration (lead), validation (equal), visualization (equal), writing – original draft (equal), writing – review and editing (equal).

## Conflicts of Interest

The authors declare no conflicts of interest.

## Data Availability

These data were derived from the following resources available in the public domain: 1. Malawi Demographic and Health Survey 2024: Key Indicators Report. https://cms.nsomalawi.mw/api/download/487/2024‐2. Malawi Demographic and Health Survey 2015‐16. https://dhsprogram.com/pubs/pdf/FR319/FR319.
